# An automated EEG–ECG framework for CAP–HRV analysis: enabling brain–heart coupling studies in sleep disorders

**DOI:** 10.3389/fphys.2026.1864664

**Published:** 2026-10-16

**Authors:** Shaween Shukir, Mostafa Moussa, Yahya AlZaabi, Ahsan Khandoker, Zbigniew R. Struzik

**Affiliations:** 1 Healthcare Engineering Innovation Group (HEIG), Department of Biomedical Engineering and Biotechnology, Khalifa University, Abu Dhabi, United Arab Emirates; 2 Graduate School of Education, University of Tokyo, Bunkyo-ku, Tokyo, Japan

**Keywords:** cyclic alternating pattern (CAP), ECG, heart rate variability (HRV), major depressive disorder, sleep, obstructive sleep apnea

## Abstract

**Background:**

Multimodal sleep analysis typically requires separate processing pipelines for electroencephalography (EEG) and electrocardiography (ECG), which complicates joint investigation of sleep microstructure and autonomic regulation. Cyclic alternating pattern (CAP) scoring and heart rate variability (HRV) analysis are routinely performed in different software environments, limiting temporal alignment, reproducibility, and scalability.

**Methods:**

We present an open-source MATLAB framework that integrates rule-based CAP A1 detection and HRV feature extraction within a single, time-synchronized workflow. The pipeline performs standardized EEG and ECG preprocessing, rule-based CAP A1 detection during slow-wave sleep (SWS) implementing the Terzano scoring criteria, R-peak detection via the Pan–Tompkins algorithm, and computation of time-domain, frequency-domain, and nonlinear HRV features by calling the open-source PhysioZoo/mhrv toolbox directly. CAP annotations are aligned with 5-minute ECG epochs through the shared polysomnography (PSG) time base. The complete code is released as an open-source repository to support reuse and extension.

**Results:**

Because HRV features are computed by calling the PhysioZoo/mhrv toolbox directly, the pipeline introduces no reimplemented HRV routine and inherits that toolbox’s validated feature definitions. CAP A1 detection is provided as a rule-based implementation of published criteria and has not yet been benchmarked against expert manual scoring. A worked example on the OSAD+ subgroup (n = 18) demonstrates pipeline output across CAP and non-CAP epochs, and a healthy-sleep A1-event HRV reference is reported from the public CAP Sleep Database (15 controls, 785 A1 epochs) as a descriptive cross-database reference.

**Conclusion:**

The framework provides a reproducible, rule-based, and modular tool for integrated EEG–ECG analysis of sleep microstructure and autonomic dynamics. By enabling synchronized CAP–HRV feature extraction in a single environment, it lowers the engineering barrier to brain–heart coupling research during sleep and provides a foundation for future extensions, including validated CAP detection, a matched-control study, a Python port, and cloud-based deployment.

## 1 Introduction

Sleep is a neuro-cardiorespiratory process in which the cerebral and autonomic systems work together to preserve homeostatic equilibrium. The cyclic alternating pattern (CAP) is a well-established EEG marker of cortical arousal and NREM sleep instability that reflects oscillations between activation (A-phases) and deactivation (B-phases) throughout the night (Terzano et al., 2001). Heart rate variability (HRV) provides a non-invasive index of autonomic tone and sympathetic–parasympathetic balance (Shaffer and Ginsberg, 2017). Converging evidence shows that moment-to-moment HRV fluctuations closely mirror EEG micro-arousals, indicating a physiological link between cortical and autonomic activity during sleep (Ferri et al., 2000; Ferini-Strambi et al., 2000). Together, CAP and HRV provide complementary insight into sleep architecture, autonomic responsiveness, and the mechanisms of arousal regulation.

Brain–heart coupling is particularly disrupted in conditions such as obstructive sleep apnea (OSA) and major depressive disorder (MDD). OSA is characterized by recurring respiratory obstructions that provoke EEG micro-arousals, sympathetic surges, and cardiovascular stress (Somers et al., 2008; Narkiewicz and Somers, 2003). CAP rates are elevated in OSA, reflecting the cortical instability driven by respiratory effort and recurrent arousals (Parrino et al., 2012). MDD is associated with poor sleep continuity, reduced slow-wave activity, and autonomic imbalance, frequently manifesting as reduced HRV and increased sympathetic tone (Benca et al., 1992; Kemp et al., 2010; Koch et al., 2019). When OSA and MDD co-occur, the combination of fragmented sleep architecture and dysregulated autonomic control may amplify physiological instability (Shukir et al., 2026).

Despite the complementary nature of EEG- and ECG-derived markers, current analytical workflows rarely integrate CAP scoring with HRV quantification in a unified environment. Manual CAP scoring remains standard but is labor-intensive, susceptible to inter-scorer variability, and difficult to scale (Rosenberg and Van Hout, 2013; Parrino et al., 2012; Mendonça et al., 2023). HRV is typically computed in separate software with independent preprocessing and manual artifact correction, complicating temporal synchronization with EEG annotations (Laborde et al., 2017). No standardized, automated pipeline currently aligns EEG-defined micro-events—specifically CAP A-phases—with beat-to-beat autonomic fluctuations (Rosenberg and Van Hout, 2013).

Recent advances in computational sleep medicine reflect a broader shift toward automated, reproducible, and scalable physiological signal processing. Deep-learning-based sleep staging and multimodal feature extraction have demonstrated that previously manual tasks can be reliably automated (Stephansen et al., 2018), reinforcing the value of open-source pipelines that standardize preprocessing and feature extraction across laboratories (Vest et al., 2018; Behar et al., 2018). Integrating EEG-derived CAP dynamics with ECG-based HRV represents a step toward precision sleep medicine, in which multimodal physiological markers enable personalized mechanistic interpretation (Topol, 2019).

Given these gaps, the present work introduces an open-source, rule-based software framework for studying brain–heart interactions during sleep. This is a Technology and Code contribution: the primary deliverable is a documented, reusable pipeline rather than a clinical study. The specific aims are: (i) to implement a modular EEG–ECG pipeline that extracts CAP dynamics and HRV features in a single, synchronized environment; (ii) to specify the synchronization logic that aligns CAP A1-phase annotations with 5-minute ECG epochs; (iii) to compute HRV features by calling the PhysioZoo/mhrv toolbox directly, so that the extracted features inherit the validated definitions of that toolbox; and (iv) to demonstrate pipeline output through a worked example on an OSA-with-comorbid-depression (OSAD+) subgroup. No clinical or statistical inference is drawn from the demonstration data.

## 2 Materials and methods

### 2.1 System architecture and software implementation

The framework is a modular, open-source system that combines EEG-based CAP detection and ECG-derived HRV computation in a single reproducible procedure. The architecture comprises five sequential modules: (i) data ingestion, (ii) signal preprocessing, (iii) CAP event detection, (iv) HRV feature extraction, and (v) multimodal synchronization and output. **Figure 1** provides a schematic overview.

**Figure 1:**
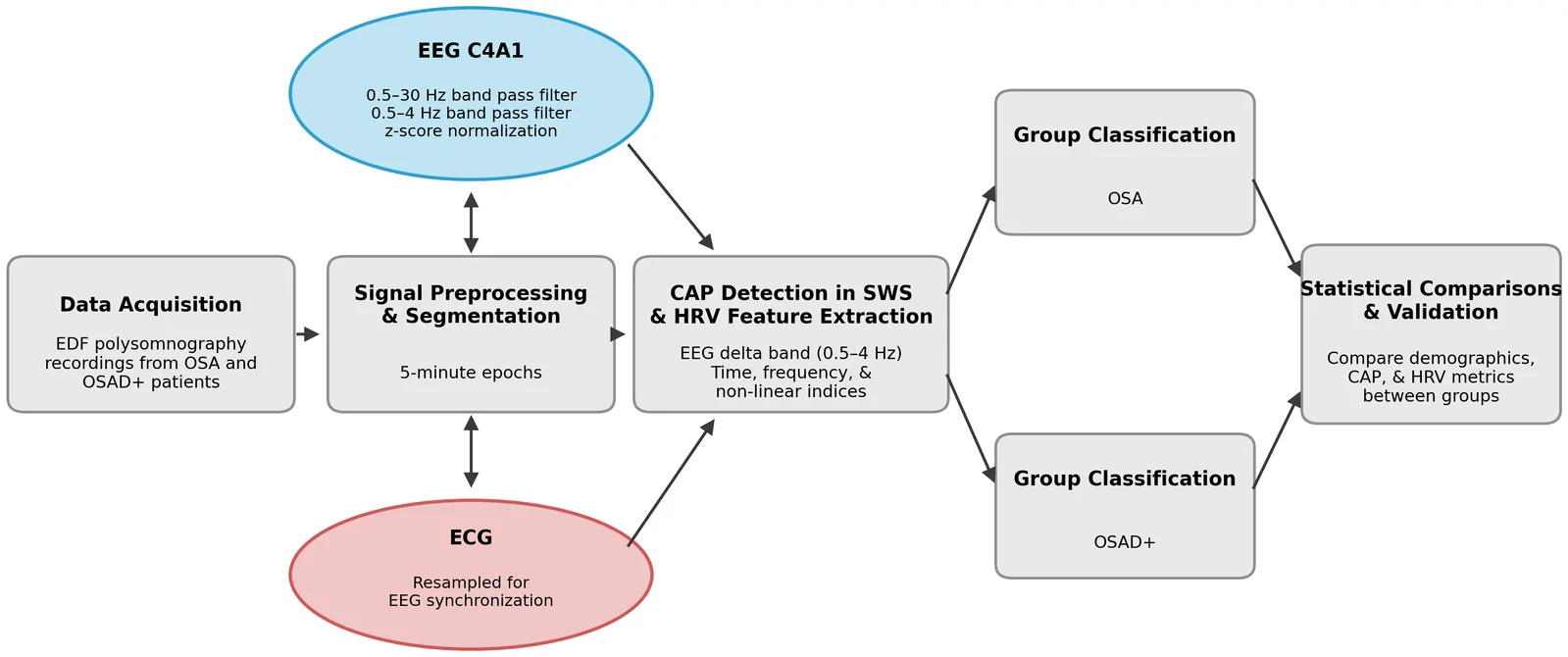
Overview of the automated EEG–ECG pipeline: PSG data acquisition, EEG and ECG preprocessing and segmentation, rule-based CAP A1 detection during slow-wave sleep, HRV feature extraction with the PhysioZoo/mhrv toolbox, synchronization of CAP annotations with 5-minute ECG epochs, and assembly of CAP-labelled HRV outputs (worked example on the OSAD+ subgroup and a descriptive control reference).

The pipeline is implemented entirely in MATLAB (R2023b; MathWorks, Natick, MA, USA). All custom scripts—covering signal preprocessing, CAP–ECG synchronization, and HRV feature extraction—are organized as independent, callable modules so that individual stages can be reused or replaced. The repository contains: (i) EEG and ECG preprocessing scripts; (ii) the CAP A1 detection module implementing the Terzano criteria (Terzano et al., 2002); (iii) the CAP–ECG synchronization module; (iv) HRV feature-extraction scripts that call the PhysioZoo/mhrv toolbox (Behar et al., 2018) directly; and (v) a worked-example script that runs the full pipeline on a representative subject. The HRV routines call the PhysioZoo/mhrv toolbox directly on the extracted R-R series, so the reported HRV values are those produced by mhrv itself. Dependencies and run instructions are documented in the repository README. The code is available at https://github.com/ahsankhandoker/CAP-HRV under a CC-BY 4.0 license, with a versioned archive deposited on Zenodo (DOI assigned on acceptance).

### 2.2 Dataset and participants

Data were obtained from the American Center for Psychiatry and Neurology (ACPN), Abu Dhabi, UAE, under Institutional Review Board approval (IRB #0019, 2017). All participants provided written informed consent. Participants were adult UAE nationals (age > 18 years) with a confirmed OSA diagnosis; patients with known cardiovascular disease were excluded. Participant characteristics are summarized in **Table 1**.

**Table 1:** Participant characteristics and PSG summary (mean ± SD unless stated).

Characteristic	OSA (n = 26)	OSAD+ (n = 18)
Age (years)	47.6 ± 13.7 (26–86)	42.9 ± 10.3 (21–62)
Sex (M/F)	17/8 (1 missing)	11/7
BMI (kg/m^2^)	36.9 ± 12.1	31.3 ± 6.3
Total Sleep Time (min)	461.6 ± 111.2	427.5 ± 123.4
Stage I (%)	8.1 ± 7.3	9.0 ± 7.1
Stage II (%)	48.4 ± 12.4	53.9 ± 15.6
Stage III (%)	15.7 ± 9.1	8.5 ± 7.2 *
REM (%)	13.9 ± 7.9	14.7 ± 9.7
Sleep Efficiency (%)	84.8 ± 13.9	85.3 ± 16.8
AHI (events/h)	29.5 ± 15.0	21.7 ± 12.7
Arousal Index	19.2 ± 12.1	18.6 ± 13.8
Mean SpO_2_ (%)	93.3 ± 2.5	88.8 ± 2.1
OSA Severity (Mild/Mod/Severe)	6/6/14	7/5/6
CAP Rate (%)	29.4 ± 23.8	34.2 ± 23.5

*p = 0.012 (Mann–Whitney U, OSA vs OSAD+).

Two patient groups were studied. The OSA-only group (n = 26; 17 male, 8 female, 1 sex not recorded; mean age 47.6 ± 13.7 years; BMI 36.9 ± 12.1 kg/m^2^) comprised patients with confirmed OSA and no comorbid depressive disorder. The OSAD+ group (n = 18; 11 male, 7 female; mean age 42.9 ± 10.3 years; BMI 31.3 ± 6.3 kg/m^2^) comprised patients with OSA and clinically assessed depressive symptoms.

Depressive symptom severity was assessed by a consultant psychiatrist using the Patient Health Questionnaire-9 (PHQ-9), a nine-item self-report instrument with total scores from 0 to 27. Published cutoffs define five severity bands: none–minimal (0–4), mild (5–9), moderate (10–14), moderately severe (15–19), and severe (20–27) (Kroenke et al., 2001). Patients scoring ≥ 10 were classified as having clinically significant depressive symptoms (PHQ-9 ≥ 10), a screening-level threshold that does not constitute a structured-interview diagnosis of MDD; this distinction is retained throughout. Patients within each group were matched by age, sex, and OSA severity.

A ten-channel PSG system (Miniscreen Plus™) was used. EEG was acquired at 100 Hz using the international 10–20 system (derivations C3-A2, C4-A1, F3-A2, F4-A1, O1-A2, O2-A1). ECG was recorded from a single Lead II configuration at 200 Hz. EOG, submental EMG, a nasal pressure transducer, thoracic/abdominal inductance belts, and pulse oximetry were also recorded. Sleep stages and respiratory events were scored according to AASM guidelines by the recording center.

### 2.3 EEG preprocessing

The C4-A1 EEG channel was selected for CAP detection, as this central derivation provides reliable detection of the high-amplitude delta-burst morphology characteristic of CAP A1 events. A 50 Hz notch filter was applied to the EEG signal to remove power-line interference, followed by a fourth-order Butterworth bandpass filter (0.5–30 Hz) spanning the delta, theta, alpha, and beta bands. A subsequent delta-band filter (0.5–4 Hz) was applied to the C4-A1 signal to isolate SWS-associated activity. Both EEG and ECG signals were standardized and segmented into consecutive, non-overlapping 5-minute epochs. Although conventional EEG scoring uses 30-second epochs, the 5-minute window was selected to provide a sufficient number of R-R intervals for stable frequency-domain and nonlinear HRV estimation, consistent with established short-term HRV recommendations (Task Force of the European Society of Cardiology and the North American Society of Pacing and Electrophysiology, 1996; Sandercock et al., 2005). The ECG signal (200 Hz) was downsampled to 100 Hz to share a common time base with the EEG for synchronization (Section 2.6); HRV features themselves were computed from R-peak timings derived at the native ECG resolution (Section 2.5).

### 2.4 CAP A1 detection (rule-based implementation)

CAP A1 events were detected using a rule-based implementation of the scoring criteria described by Terzano et al. (2002). We emphasize at the outset that this module is a deterministic implementation of published rules, not a CAP detector validated against expert manual scoring; no manually scored CAP labels were available in the present dataset, so detection performance (sensitivity, specificity, F1, Cohen’s κ) is not reported here. A validation protocol against an expert-annotated public resource is described in Section 4 as the immediate next step.

Within each 5-minute epoch, candidate CAP A1 events—high-amplitude delta bursts during SWS—were identified using the following explicit parameters, in accordance with Terzano et al. (2002): (i) minimum A-phase duration 1 s; (ii) maximum A-phase duration 60 s; (iii) amplitude criterion—the peak delta-band envelope within the candidate event must exceed the mean background delta-band amplitude of the surrounding epoch by a factor of at least 1.33 (i.e., ≥ 1/3 above background); and (iv) the candidate event must fall within a polysomnographically confirmed Stage III (slow-wave) sleep epoch. The background amplitude reference was the mean absolute value of the delta-filtered C4-A1 signal over the full 5-minute epoch, excluding the candidate-event window. The current implementation detects CAP A1 subtypes during SWS only; CAP A2/A3 subtypes and CAP occurring during Stage 2 NREM—both clinically relevant, particularly in OSA, where A3 events predominate—are outside the present scope and are noted as planned extensions (Section 4).

### 2.5 ECG preprocessing and HRV feature extraction

ECG epochs corresponding to CAP and non-CAP periods were processed separately. R-peaks were detected with the Pan–Tompkins algorithm (Pan and Tompkins, 1985). A multi-step fallback strategy handled epochs with poor peak detection: if fewer than five R-peaks were detected, the detector was re-applied at a reduced effective sampling rate, then after discarding the first 500 samples of the epoch, and finally on an amplitude-clipped version of the signal. Epochs yielding fewer than five R-peaks after all fallback steps were discarded. R-R intervals were computed as the time difference between consecutive R-peaks. Ectopic and artifactual intervals were screened at the epoch level: epochs with a mean heart rate below 50 bpm or an SDNN above 300 ms were excluded as physiologically implausible, consistent with standard short-term HRV quality control (Task Force of the European Society of Cardiology and the North American Society of Pacing and Electrophysiology, 1996; Laborde et al., 2017). No interpolation or resampling of the R-R series was applied prior to nonlinear analysis. The number and proportion of retained versus excluded epochs are reported in Section 3.1, with the per-group breakdown of excluded epochs to allow assessment of selection bias.

HRV features were computed by calling the PhysioZoo/mhrv toolbox directly. The extracted features (defined in **Table 2**) comprise mean heart rate (HR); time-domain indices (AVNN, SDNN, RMSSD, pNN50, SEM); frequency-domain indices computed via Lomb–Scargle spectral analysis (VLF, LF, HF, total power, nVLF, nLF, nHF, LF/HF, LF peak, HF peak); nonlinear indices (SD1, SD2, DFA α1, DFA α2, SampEn); and the spectral slope β — 22 features in total.

**Table 2:** Definitions and physiological relevance of the 22 HRV features extracted from ECG epochs.

Domain	Feature	Definition	Physiological significance
Heart rate	HR (bpm)	Mean heart rate over the epoch (60,000/AVNN).	Overall cardiac rate; elevated with sympathetic activation.
Time	AVNN (ms)	Mean of all normal-to-normal (NN) intervals.	Average heart period; lower values indicate higher sympathetic drive.
	SDNN (ms)	Standard deviation of all NN intervals.	Overall HRV and total autonomic input.
	RMSSD (ms)	Root mean square of successive NN differences.	Short-term beat-to-beat variation; primarily parasympathetic.
	pNN50 (%)	Percentage of successive NN intervals differing by more than 50 ms.	Established index of vagal (parasympathetic) tone.
	SEM (ms)	Standard error of the mean NN interval.	Signal stability within an epoch.
Frequency	VLF (ms²)	Spectral power within 0.003–0.04 Hz (Lomb–Scargle).	Thermoregulation, vasomotor tone, renin–angiotensin activity.
	LF (ms²)	Spectral power within 0.04–0.15 Hz.	Mixed sympathetic and parasympathetic modulation.
	HF (ms²)	Spectral power within 0.15–0.4 Hz.	Respiratory-linked parasympathetic modulation.
	Total power (ms²)	Total spectral power across the VLF, LF, and HF bands.	Overall autonomic modulation.
	nVLF (%)	Normalized VLF power (percentage of VLF+LF+HF power).	Scale-invariant share of very-low-frequency power.
	nLF (%)	Normalized LF power (percentage of VLF+LF+HF power).	Scale-invariant LF contribution (note: nVLF+nLF+nHF ≈ 100%).
	nHF (%)	Normalized HF power (percentage of VLF+LF+HF power).	Scale-invariant HF contribution.
	LF/HF	Ratio of low- to high-frequency power.	Index of sympathovagal balance.
	LF peak (Hz)	Frequency of the dominant spectral peak within the LF band.	Characteristic LF oscillation frequency.
	HF peak (Hz)	Frequency of the dominant spectral peak within the HF band.	Characteristic HF (respiratory) oscillation frequency.
Nonlinear	SD1 (ms)	Short-axis SD of the Poincaré plot.	Short-term variability related to vagal activity.
	SD2 (ms)	Long-axis SD of the Poincaré plot.	Overall variability including both autonomic branches.
	DFA α1	Short-term fractal scaling exponent (4–16 beats).	Short-range RR correlations.
	DFA α2	Long-term fractal scaling exponent (16–64 beats).	Long-range RR correlations and complexity.
	SampEn	Sample entropy of the RR series.	Signal complexity; lower values indicate more regular rhythms.
Spectral slope	β (slope)	Slope of the log–log Lomb–Scargle power spectrum.	1/f-like scaling of HRV dynamics.

### 2.6 CAP–ECG synchronization

EEG-derived CAP annotations and ECG-derived HRV features were integrated at the epoch level through the shared PSG time base. The procedure is as follows. First, NREM intervals identified from the hypnogram were divided into consecutive 5-minute epochs. Sleep staging is performed on standard 30-second PSG epochs; a 5-minute analysis epoch therefore spans ten consecutive 30-second staged epochs. A 5-minute epoch was admitted to CAP–HRV analysis only if it lay within a contiguous NREM segment, and the Stage III requirement of the CAP A1 rule (Section 2.4) was evaluated against the underlying 30-second staging: a candidate A1 event was accepted only when its supporting 30-second epoch was scored Stage III. Thus the 5-minute window defines the HRV analysis length, while the 30-second staging governs CAP eligibility; the two are not conflated.

Second, for each admitted epoch, the CAP A1 detector (Section 2.4) determined whether at least one qualifying A1 event was present. The epoch was labeled CAP (1) if it contained one or more A1 events and non-CAP (0) otherwise; epochs containing a mixture of CAP and non-CAP intervals were labeled CAP on the presence of any qualifying A1 event. Third, the corresponding 5-minute ECG segment was extracted using the shared timestamp, and HRV features were computed from it (Section 2.5), producing one feature vector per epoch. The final output is a synchronized table in which each row is one 5-minute epoch with a full HRV feature vector and a CAP label.

This design has an important and explicit consequence for interpretation. A CAP A1 event lasts 1–60 s, whereas the analysis epoch is 300 s; a single qualifying event therefore occupies at most 20% and frequently far less of the labeled epoch. The measured HRV consequently reflects the entire epoch—both arousal-associated and inter-arousal intervals—rather than the instantaneous autonomic response to an individual A-phase. Because the present dataset stored CAP information at the epoch and patient level rather than as event-level onset/offset timings, the exact fraction of each epoch occupied by A1 activity could not be recomputed here; it is therefore reported descriptively at the patient level as the CAP rate (**Table 1**) and is flagged as a quantity to be measured directly in the validation work described in Section 4. The released synchronization module exposes the event-level timing hooks required for an event-locked analysis at finer temporal resolution, which we identify as the appropriate route for isolating event-related autonomic effects. **Figure 2** depicts the temporal mapping of CAP events to ECG epochs.

**Figure 2:**
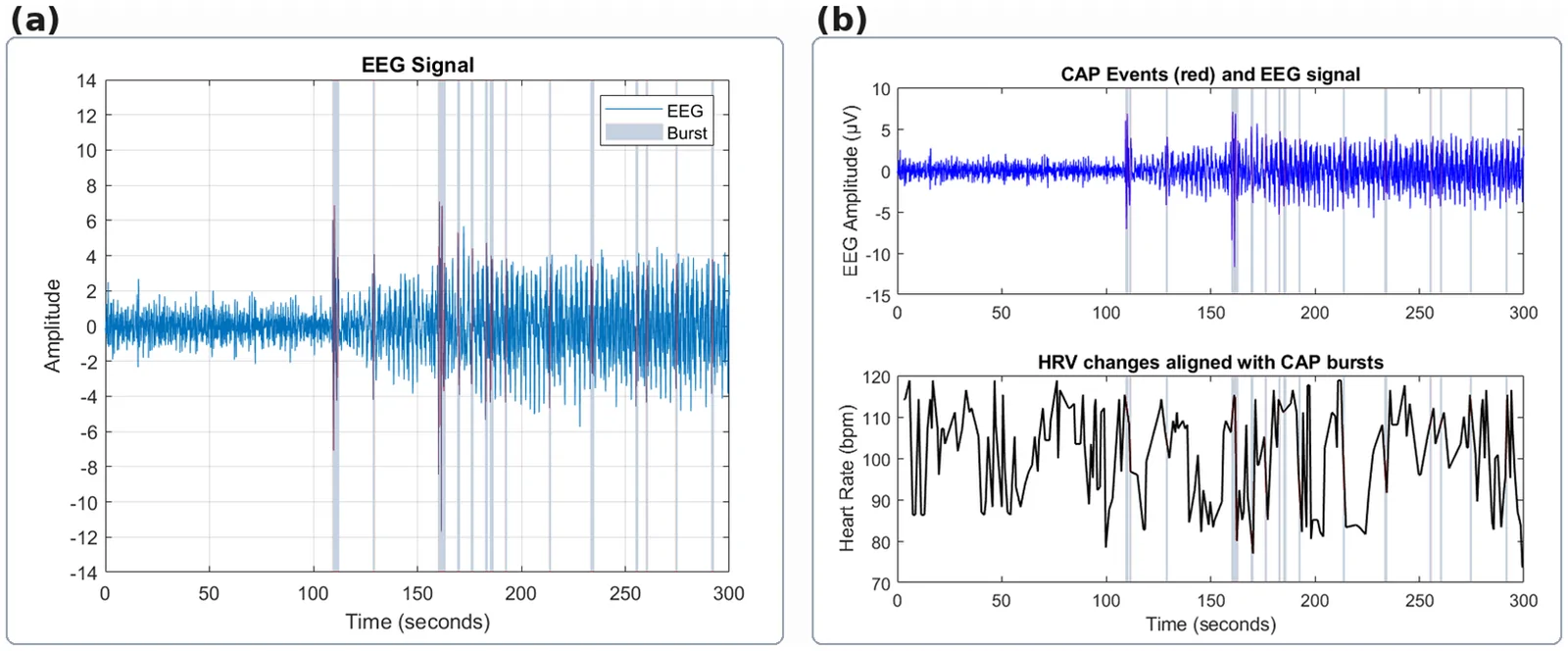
CAP–ECG temporal mapping for HRV feature computation. **(A)** the EEG signal with shaded CAP A1 bursts, and **(B)** the EEG signal with CAP A1 event markers (top) together with the aligned instantaneous heart rate (bottom).

### 2.7 Reproducibility

The pipeline contains no stochastic components: the bandpass and notch filters are linear time-invariant operations, the Pan–Tompkins detector is threshold-based and deterministic, the Lomb–Scargle estimator operates on fixed-length R-R arrays without random initialization, and the nonlinear routines (DFA, SampEn, Poincaré) are deterministically defined. The pipeline is therefore deterministic by construction and returns identical outputs for identical inputs within a fixed software environment; we report this as a design property rather than as an empirical result, since re-running deterministic code on fixed hardware is expected to reproduce its outputs. A more informative robustness check—quantifying output variation across operating systems and MATLAB versions—is identified as future work in Section 4.

### 2.8 Control reference cohort and A1-event HRV

To provide a healthy-sleep HRV reference for CAP A1 events, we additionally analyzed the publicly available CAP Sleep Database (capslpdb) (Goldberger et al., 2000; Terzano et al., 2001), a collection of expert-scored polysomnographic recordings from the Sleep Disorders Center of the Ospedale Maggiore of Parma, Italy. The database provides 16 healthy controls (records n1–n16); one recording (n16) contains EEG only and lacks an ECG channel and was therefore excluded, leaving 15 controls with ECG. For each control, the 5-minute ECG epochs coinciding with expert-annotated CAP A1 events during NREM sleep were extracted (810 A1 epochs in total) and processed with the same HRV pipeline used for the patient data (Sections 2.4–2.5): R-peaks were detected with the Pan–Tompkins algorithm, epochs failing the quality criteria of Section 2.5 were discarded, the full HRV feature set was computed per epoch, and per-epoch values were averaged within each subject. This cohort is used strictly as a descriptive, cross-database HRV reference; because it differs from the patient recordings in database, site, acquisition system, and population, no statistical comparison between controls and patients is performed (Section 4).

## 3 Results

### 3.1 Pipeline performance and epoch retention

The integrated framework automated all processing steps—CAP detection, R-peak extraction, HRV computation, and feature assembly—end to end. On the test workstation (Windows 10 x64, Intel Core i7, 16 GB RAM), runtime profiling yielded a mean processing time of approximately 12 s per epoch, corresponding to 8–10 min per subject for a full overnight recording. For reference, manual CAP scoring combined with separate HRV extraction typically requires 20–30 min per subject (Berntson et al., 2005; Parrino et al., 2012; Hartmann and Baumert, 2019; Vest et al., 2018).

Epoch retention after quality filtering varied across subjects. On average, 50.5% of NREM epochs passed the quality thresholds (heart rate ≥ 50 bpm and SDNN ≤ 300 ms), corresponding to a mean of 29.1 ± 21.7 retained epochs per subject across the OSA and OSAD+ groups combined; 43 of 44 subjects yielded at least one valid epoch. Because this exclusion rate is substantial, we examined whether retention was systematically related to signal quality. Excluded epochs were dominated by low R-peak yield and high-SDNN artifact (noise-contaminated segments) rather than by physiological bradycardia: the 50 bpm threshold targets noise rather than sleep bradycardia, which was not observed as a systematic feature of this dataset. We note as a limitation (Section 4) that a fuller characterization of retained versus excluded epochs by sleep-stage distribution and time-of-night would further constrain potential selection bias; the released pipeline logs the per-epoch exclusion reason to support this in future analyses.

### 3.2 Worked example: running the pipeline end to end

To illustrate typical use, the repository includes a worked-example script that ingests a single subject’s EEG and ECG, runs preprocessing, CAP A1 detection, R-peak detection, HRV extraction, and synchronization, and writes the synchronized feature table. On a representative OSAD+ subject the script produced a labeled table of 5-minute epochs, each carrying the full HRV feature vector and its CAP label, with the per-epoch exclusion log written alongside. This example serves as the entry point for new users and as a template for batch processing across a cohort. Practical limitations of the example—single-channel CAP detection, offline (non-real-time) operation, and MATLAB licensing—are discussed in Section 4.

### 3.3 Reproducibility

Consistent with the deterministic design described in Section 2.7, repeated executions of the complete pipeline on identical input data under a fixed software environment produced numerically identical HRV feature vectors at floating-point precision. We report this as confirmation of deterministic behavior rather than as evidence of cross-environment robustness; quantifying variation across operating systems and MATLAB versions remains future work (Section 4).

### 3.4 Descriptive case study: OSAD+ subgroup

To illustrate pipeline output on real physiological data, the framework was applied to the OSAD+ subgroup (n = 18). This analysis is strictly a technical demonstration of output structure; no statistical or clinical inference is drawn from it. Descriptive summaries of extracted HRV features for CAP and non-CAP epochs are presented in **Table 3**. The pipeline produced complete, well-structured feature vectors for every retained epoch, with values falling in physiologically plausible ranges. We deliberately refrain from interpreting CAP-versus-non-CAP differences in this subgroup as physiological findings, because (i) no inferential test is applied and (ii) the epoch-level labeling described in Section 2.6 means any such difference reflects epoch context rather than event-locked autonomic response.

**Table 3:** Descriptive summary of HRV measures for CAP and non-CAP epochs in the OSAD+ demonstration group (n = 18; mean ± SEM).

HRV measure	CAP epochs (mean ± SEM)	Non-CAP epochs (mean ± SEM)
HR (bpm)	83.74 ± 6.18	71.86 ± 2.71
AVNN (ms)	778.50 ± 42.52	864.70 ± 28.07
SDNN (ms)	102.50 ± 19.55	76.32 ± 15.66
RMSSD (ms)	97.14 ± 27.81	66.17 ± 16.86
pNN50 (%)	90.85 ± 1.72	90.57 ± 1.34
SEM (ms)	5.08 ± 0.84	5.15 ± 1.70
β (spectral slope)	−0.63 ± 0.08	−0.54 ± 0.06
LF peak (Hz)	0.07 ± 0.00	0.07 ± 0.00
HF peak (Hz)	0.26 ± 0.01	0.26 ± 0.01
LF (ms^2^)	2463 ± 612	4138 ± 1973
HF (ms^2^)	3955 ± 1490	8641 ± 4573
VLF (ms^2^)	4377 ± 961	4188 ± 1213
nHF (%)	26.43 ± 3.08	25.66 ± 2.29
nLF (%)	25.61 ± 1.44	28.77 ± 1.52
nVLF (%)	44.99 ± 2.77	42.80 ± 1.69
LF/HF	1.98 ± 0.43	2.53 ± 0.53
Total Power (ms^2^)	11094 ± 2772	17243 ± 7677
SD1 (ms)	68.92 ± 19.66	47.74 ± 12.63
SD2 (ms)	123.40 ± 20.32	93.75 ± 17.46
DFA α1	1.00 ± 0.06	1.05 ± 0.04
DFA α2	0.94 ± 0.03	0.89 ± 0.02
SampEn	1.00 ± 0.05	1.02 ± 0.02

No inferential statistics are applied; this table illustrates pipeline output only.

### 3.5 Control A1-event HRV (reference cohort)

Applying the pipeline to the 15 capslpdb control subjects, 785 of 810 A1 epochs (96.9%) passed quality control—substantially higher retention than in the patient recordings (Section 3.1), consistent with the cleaner signals of healthy volunteers. The resulting A1-event HRV is summarized in **Table 4**. The values are physiologically consistent with a young, healthy cohort (ages 23–42 years): mean heart rate 65.2 ± 6.8 bpm, AVNN 933.8 ± 102.0 ms, and comparatively high vagally mediated variability (RMSSD 82.8 ± 35.6 ms; pNN50 95.4 ± 3.6%, computed with the same threshold convention as the patient pipeline), with sympathovagal balance below unity (LF/HF 0.79 ± 0.21) and fractal and entropy indices in the expected ranges (DFA α1 0.81 ± 0.10; SampEn 1.68 ± 0.21). Internal consistency checks confirmed that SD1 reproduced RMSSD/√2 to within 0.5% and that nLF + nHF summed to 100% in every subject. In this cohort the 50 bpm exclusion rarely bound: retained epochs had a median heart rate of 65 bpm (5th–95th percentile 52–77 bpm), only 10.6% fell below 55 bpm, and none of the 25 excluded epochs were excluded for physiological bradycardia (exclusions were dominated by low R-peak yield), supporting the cutoff as a noise rather than a bradycardia filter. These values are provided as a healthy-sleep A1-event HRV reference only; because they derive from a different database, site, and acquisition system than the patient recordings, they are not compared statistically with the OSA or OSAD+ groups (Section 4).

**Table 4:** Control reference cohort — full HRV feature set over CAP A1-event epochs (capslpdb healthy controls, n = 15; 796 A1 epochs; mean ± SD across subjects; HRV computed with the PhysioZoo/mhrv toolbox).

HRV measure	Control A1 epochs (mean ± SD, n = 15)
HR (bpm)	66.6 ± 7.5
AVNN (ms)	919.3 ± 106.4
SDNN (ms)	72.1 ± 33.7
RMSSD (ms)	70.7 ± 43.1
pNN50 (%)	29.8 ± 21.7
SEM (ms)	4.1 ± 2.0
VLF (ms²)	2242 ± 1845
LF (ms²)	1138 ± 915
HF (ms²)	1674 ± 1761
Total power (ms²)	5193 ± 4261
nVLF (%)	31.6 ± 5.0
nLF (%)	28.3 ± 4.0
nHF (%)	38.3 ± 7.3
LF/HF	1.18 ± 0.49
LF peak (Hz)	0.072 ± 0.008
HF peak (Hz)	0.273 ± 0.038
SD1 (ms)	50.1 ± 30.5
SD2 (ms)	86.8 ± 37.2
DFA α1	0.88 ± 0.11
DFA α2	0.76 ± 0.06
SampEn	1.58 ± 0.16
β (slope)	−0.35 ± 0.21

Reported descriptively as a cross-database reference; no statistical comparison with the patient groups is performed.

## 4 Discussion

This paper presents a rule-based, modular, and deterministic MATLAB framework for integrated EEG–ECG analysis of sleep microstructure and autonomic dynamics. The pipeline automates EEG and ECG preprocessing, implements Terzano-criterion CAP A1 detection during SWS, synchronizes CAP annotations with 5-minute ECG epochs through a shared PSG time base, and extracts a comprehensive HRV feature set spanning time, frequency, and nonlinear domains. Its contribution is engineering and methodological: it lowers the barrier to synchronized CAP–HRV feature extraction, which previously required two separate software environments and manual temporal alignment.

A distinction must be drawn between the two analytic components. HRV features are produced by the PhysioZoo/mhrv toolbox invoked directly, so they inherit that toolbox’s validated definitions and are not reimplemented or re-benchmarked here. The CAP detection module, by contrast, is a rule-based implementation of published criteria and has not been benchmarked against expert manual scoring; no manually scored CAP labels were available in this dataset. Accordingly, we do not describe the CAP component as validated. The immediate next step—and the most important one for this pipeline—is to benchmark CAP A1 detection against an expert-annotated public resource such as the CAP Sleep Database on PhysioNet, reporting sensitivity, specificity, F1, and Cohen’s κ against the manual scores; the released code is structured to accept that database’s annotations directly, and a validation protocol accompanies the repository.

A key methodological point concerns the CAP-to-ECG epoch mapping. CAP A1 events last 1–60 s, yet stable HRV estimation requires windows of at least several minutes. Labeling a 5-minute epoch as CAP whenever it contains any A1 event means the measured HRV reflects the whole epoch, and brief events are diluted within it; a single event can occupy as little as a fraction of one percent of the window. The CAP-versus-non-CAP HRV contrasts reported here—and many comparable contrasts in the literature (Ferri et al., 2000; Ferini-Strambi et al., 2000; Somers et al., 1993)—therefore characterize epoch-level autonomic context, not the instantaneous response to individual arousals. Researchers requiring event-locked HRV (e.g., in the seconds surrounding each A-phase) should use the event-level timing hooks exposed by the synchronization module; quantifying per-epoch A1 occupancy and testing whether autonomic differences scale with within-epoch CAP density are the natural first analyses on that path. The closest methodological precedent for such event-locked CAP–HRV analysis is the work of Ferri and colleagues (Ferri et al., 2000; Ferini-Strambi et al., 2000), who characterized autonomic activation during CAP A-phases at fine temporal resolution; the planned event-locked mode would extend that approach within the present integrated, open-source pipeline.

Several limitations constrain the current implementation. First, CAP detection was not benchmarked against expert scorers, and detects only A1 subtypes during SWS; A2/A3 subtypes and Stage 2 CAP are not yet implemented. Second, the control reference cohort (Sections 2.8 and 3.5) is a descriptive cross-database reference, not a matched control arm: it derives from a different database (capslpdb; Parma, Italy), site, and acquisition system, and a younger, healthy population (ages 23–42 years), and lacks patient-matched demographic and PSG data. Any control-versus-patient contrast would therefore confound group status with database, site, equipment, and age; for this reason no between-group statistical comparison is performed, and a matched, single-site control arm remains necessary before any control-referenced claim can be made. Third, the MATLAB dependency limits accessibility for researchers without a license. Fourth, all patients are from a single UAE institution, limiting generalizability. Fifth, the pipeline is offline only and has not been evaluated for real-time use. Sixth, the 50 bpm heart-rate exclusion, while consistent with HRV quality-control conventions, may occasionally exclude valid bradycardic epochs in specific populations, though this was not observed here. Seventh, the PHQ-9 ≥ 10 criterion is a screening-level threshold, not a structured-interview diagnosis of MDD.

Two points follow for interpretation. First, the principal contribution of this work is the open-source CAP–HRV pipeline, demonstrated on the OSA and OSAD+ recordings; the control-group analysis is provided only as an exploratory HRV reference, and the conclusions of the manuscript do not rely on it. Second, the control sample was available primarily as an HRV reference cohort and was not fully phenotyped with the same demographic and polysomnographic detail as the OSA cohort; consequently, comparisons involving controls should be interpreted as exploratory and hypothesis-generating rather than as baseline-matched group comparisons.

The framework is designed for scalability. Its modular structure allows individual stages to be parallelized or replaced; the per-epoch processing model maps naturally onto batch execution across large sleep databases; and the deterministic design makes distributed runs auditable. Planned developments include: benchmarking CAP detection against the PhysioNet CAP Sleep Database with standard performance metrics; extending detection to CAP A2/A3 and Stage 2 NREM; porting the pipeline to Python to remove the licensing barrier and enable cloud deployment; adding an event-locked synchronization mode; and evaluating robustness across operating systems and MATLAB versions. Compared with existing tools—Kubios (proprietary; HRV only), PhysioZoo (open-source; HRV only) (Behar et al., 2018; Vest et al., 2018), and manual CAP scoring tools (labor-intensive; variable) (Rosenberg and Van Hout, 2013; Parrino et al., 2012; Mendonça et al., 2023)—the present framework fills the specific niche of synchronized, automated, open-source CAP–HRV feature extraction in a single environment, while being explicit about what it does and does not yet validate. Additional autonomic context for CAP and arousal-related HRV changes is provided by prior physiological studies (Somers et al., 1993; Narkiewicz et al., 1998; Licht et al., 2008; Kemp et al., 2010; Narkiewicz and Somers, 2003).

## 5 Conclusion

We present an open-source EEG–ECG framework for extracting integrated CAP and HRV features from overnight polysomnography within a single, time-synchronized environment. The pipeline implements rule-based CAP A1 detection during SWS using Terzano criteria, synchronizes annotations with 5-minute ECG epochs through a shared PSG time base, and extracts a comprehensive HRV feature set by calling the PhysioZoo/mhrv toolbox directly. The pipeline is deterministic by construction. A worked example and a descriptive OSAD+ case study demonstrate output structure across CAP and non-CAP epochs. The CAP component is provided as a rule-based implementation pending validation against expert scoring, which we identify as the principal next step. The framework provides an accessible, modular foundation for multimodal sleep research on brain–heart interactions and for future extensions including validated CAP detection, a Python port, and cloud-based deployment.

## Data Availability

The original contributions presented in the study are included in the article/supplementary material. Further inquiries can be directed to the corresponding author. The pipeline source code and demo dataset are available at https://github.com/ahsankhandoker/CAP-HRV and the control data are the public CAP Sleep Database (capslpdb) on PhysioNet.

## References

[B1] Behar J. A. Rosenberg A. A. Weiser-Bitoun I. Shemla O. Alexandrovich A. Konyukhov E. . (2018). PhysioZoo: a novel open-access platform for heart rate variability analysis of mammalian electrocardiographic data. Front. Physiol. 9. doi: 10.3389/fphys.2018.01390 30337883 PMC6180147

[B2] Benca R. M. Obermeyer W. H. Thisted R. A. Gillin J. C. (1992). Sleep and psychiatric disorders: a meta-analysis. Arch. Gen. Psychiatry 49, 651–668. doi: 10.1001/archpsyc.1992.01820080059010 1386215

[B3] Berntson G. G. Lozano D. L. Chen Y.-J. (2005). Filter properties of root mean square successive difference (RMSSD) for heart rate. Psychophysiology 42, 246–252. doi: 10.1111/j.1469-8986.2005.00277.x 15787862

[B4] Ferini-Strambi L. Bianchi A. Zucconi M. Oldani A. Castronovo V. Smirne S. (2000). The impact of cyclic alternating pattern on heart rate variability during sleep in healthy young adults. Clin. Neurophysiol. 111, 99–101. doi: 10.1016/S1388-2457(99)00212-6 10656516

[B5] Ferri R. Parrino L. Smerieri A. Terzano M. G. Elia M. Musumeci S. A. . (2000). Cyclic alternating pattern and spectral analysis of heart rate variability during normal sleep. J. Sleep Res. 9, 13–18. doi: 10.1046/j.1365-2869.2000.00190.x 10733684

[B6] Goldberger A. L. Amaral L. A. N. Glass L. Hausdorff J. M. Ivanov P. C. Mark R. G. . (2000). PhysioBank, PhysioToolkit, and PhysioNet: components of a new research resource for complex physiologic signals. Circulation 101, e215–e220. doi: 10.1161/01.CIR.101.23.e215 10851218

[B7] Hartmann S. Baumert M. (2019). Automatic A-phase detection of cyclic alternating patterns in sleep using dynamic temporal information. IEEE Trans. Neural Syst. Rehabil. Eng. 27, 1695–1703. doi: 10.1109/TNSRE.2019.2934828 31425039

[B8] Kemp A. H. Quintana D. S. Gray M. A. Felmingham K. L. Brown K. Gatt J. M. (2010). Impact of depression and antidepressant treatment on heart rate variability: a review and meta-analysis. Biol. Psychiatry 67, 1067–1074. doi: 10.1016/j.biopsych.2009.12.012 20138254

[B9] Koch C. Wilhelm M. Salzmann S. Rief W. Euteneuer F. (2019). A meta-analysis of heart rate variability in major depression. Psychol. Med. 49, 1948–1957. doi: 10.1017/S0033291719001351 31239003

[B10] Kroenke K. Spitzer R. L. Williams J. B. W. (2001). The PHQ-9: validity of a brief depression severity measure. J. Gen. Intern. Med. 16, 606–613. doi: 10.1046/j.1525-1497.2001.016009606.x 11556941 PMC1495268

[B11] Laborde S. Mosley E. Thayer J. F. (2017). Heart rate variability and cardiac vagal tone in psychophysiological research — recommendations for experiment planning, data analysis, and data reporting. Front. Psychol. 8, 213. doi: 10.3389/fpsyg.2017.00213 28265249 PMC5316555

[B12] Licht C. M. M. de Geus E. J. C. Zitman F. G. Hoogendijk W. J. G. van Dyck R. Penninx B. W. J. H. (2008). Association between major depressive disorder and heart rate variability in the Netherlands Study of Depression and Anxiety (NESDA). Arch. Gen. Psychiatry 65, 1358–1367. doi: 10.1001/archpsyc.65.12.1358 19047522

[B13] Mendonça F. Mostafa S. S. Morgado-Dias F. Ravelo-García A. G. Rosenzweig I. (2023). Towards automatic EEG cyclic alternating pattern analysis: a systematic review. Biomed. Eng. Lett. 13, 273–291. doi: 10.1007/s13534-023-00303-w 37519874 PMC10382419

[B14] Narkiewicz K. Montano N. Cogliati C. van de Borne P. J. Dyken M. E. Somers V. K. (1998). Altered cardiovascular variability in obstructive sleep apnea. Circulation 98, 1071–1077. doi: 10.1161/01.CIR.98.11.1071 9736593

[B15] Narkiewicz K. Somers V. K. (2003). Sympathetic nerve activity in obstructive sleep apnoea. Acta Physiol. Scand. 177, 385–390. doi: 10.1046/j.1365-201X.2003.01091.x 12609010

[B16] Pan J. Tompkins W. J. (1985). A real-time QRS detection algorithm. IEEE Trans. Biomed. Eng. 32, 230–236. doi: 10.1109/TBME.1985.325532 3997178

[B17] Parrino L. Ferri R. Bruni O. Terzano M. G. (2012). Cyclic alternating pattern (CAP): the marker of sleep instability. Sleep Med. Rev. 16, 27–45. doi: 10.1016/j.smrv.2011.02.003 21616693

[B18] Rosenberg R. S. Van Hout S. (2013). The American Academy of Sleep Medicine inter-scorer reliability program: sleep stage scoring. J. Clin. Sleep Med. 9, 81–87. doi: 10.5664/jcsm.2350 23319910 PMC3525994

[B19] Sandercock G. R. H. Bromley P. D. Brodie D. A. (2005). The reliability of short-term measurements of heart rate variability. Int. J. Cardiol. 103, 238–247. doi: 10.1016/j.ijcard.2004.09.013 16098384

[B20] Shaffer F. Ginsberg J. P. (2017). An overview of heart rate variability metrics and norms. Front. Public Health 5. doi: 10.3389/fpubh.2017.00258 29034226 PMC5624990

[B21] Shukir S. Khandoker A. H. Moussa M. Chowdhury N. Struzik Z. R. (2026). Sample entropy of heart rate variability during cyclic alternating pattern in obstructive sleep apnoea with and without comorbid depression. Eur. Phys. J. Spec. Top. doi: 10.1140/epjs/s11734-026-02555-w

[B22] Somers V. K. Dyken M. E. Mark A. L. Abboud F. M. (1993). Sympathetic-nerve activity during sleep in normal subjects. N. Engl. J. Med. 328, 303–307. doi: 10.1056/NEJM199302043280502 8419815

[B23] Somers V. K. White D. P. Amin R. Abraham W. T. Costa F. Culebras A. . (2008). Sleep apnea and cardiovascular disease: an American Heart Association/American College of Cardiology Foundation scientific statement. Circulation 118, 1080–1111. doi: 10.1016/j.jacc.2008.05.002 18725495

[B24] Stephansen J. B. Olesen A. N. Olsen M. Ambati A. Leary E. B. Moore H. E. . (2018). Neural network analysis of sleep stages enables efficient diagnosis of narcolepsy. Nat. Commun. 9, 5229. doi: 10.1038/s41467-018-07229-3 30523329 PMC6283836

[B25] Task Force of the European Society of Cardiology and the North American Society of Pacing and Electrophysiology (1996). Heart rate variability: standards of measurement, physiological interpretation, and clinical use. Circulation 93, 1043–1065. doi: 10.1161/01.CIR.93.5.1043 8598068

[B26] Terzano M. G. Parrino L. Sherieri A. Chervin R. Chokroverty S. Guilleminault C. . (2001). Atlas, rules, and recording techniques for the scoring of cyclic alternating pattern (CAP) in human sleep. Sleep Med. 2, 537–553. doi: 10.1016/S1389-9457(01)00149-6 14592270

[B27] Terzano M. G. Parrino L. Smerieri A. Chervin R. Chokroverty S. Guilleminault C. . (2002). Atlas, rules, and recording techniques for the scoring of cyclic alternating pattern (CAP) in human sleep (revised). Sleep Med. 3, 187–199. doi: 10.1016/S1389-9457(02)00003-5 14592244

[B28] Topol E. J. (2019). Deep Medicine: How Artificial Intelligence Can Make Healthcare Human Again (New York: Basic Books).

[B29] Vest A. N. Da Poian G. Li Q. Liu C. Nemati S. Shah A. . (2018). An open-source benchmarked toolbox for cardiovascular waveform and interval analysis. Physiol. Meas. 39, 105004. doi: 10.1088/1361-6579/aae021 30199376 PMC6442742

